# P09-08 24-Hour Physical Behavior Balance for Better Health for All: “The Sweet-Spot Hypothesis”

**DOI:** 10.1093/eurpub/ckac095.138

**Published:** 2022-08-29

**Authors:** Nidhi Gupta, Bart Cillekens, Margo Ketels, David Hallman, Els Clays, Huysmans Maaike, Holtermann Andreas, Pieter Coenen

**Affiliations:** 1 National Research Centre for the Working Environment, National Research Centre for the Working Environment, Copenhagen, Denmark; 2 Department of Public and Occupational Health, AmsterdamUMC, Amsterdam, The Netherlands; 3 Department of Public Health and Primary Care, Ghent University, Gent, Belgium; 4 Department of Occupational and Public Health Sciences, University of Gävle, Gavle, Sweden

**Keywords:** Physical activity domains - Work - Leisure time - Sleep - Sedentary behaviour

## Abstract

**Background:**

‘Sit less-move more' has been a common advice to improve health of adults. Research indicates that this advice might be health enhancing among adults with sedentary occupations but not among adults with physically active occupations such as cleaners. This may be explained by the considerable differences in 24-h physical behaviors between adults in sedentary and physically active occupations. To provide a scientific approach and encourage research on 24-h physical behaviors and health for those in physically active occupations, we recently proposed the ‘Sweet-Spot Hypothesis'. The hypothesis postulated that the ‘Sweet-Spot' of 24-h physical behaviors for better health differs between adults, depending on their occupation.

**Methods:**

To exemplify such hypothesis, we tested the cross-sectional association between 24-hour time composition of physical behaviors measured using thigh-based accelerometry and self-rated health among adults engaged in white-collar (n = 136), manufacturing (n = 481) and cleaning (n = 130) occupations.

**Results:**

We found that the sweet spot of 24-h physical behaviors for better health was far from ‘sit less-move more' zone among adults with physically active occupations. Specifically, among white-collar workers, 24-h physical behavior distribution associated with the best 5% of self-rated health comprised about 30% of the day spent on sedentary behavior, 45% spent actively, and 25% spent on sleep. However, among cleaners, this distribution was about 50% spent sedentary, 15% spent actively, and 35% on sleep and in manufacturing sector, this distribution was about 35% spent sedentary, 35% spent actively, and 30% spent on sleep.

**Conclusion:**

The advice ‘sit less-move more' may not bring adults in physically active occupations toward their ‘Sweet-Spot' of 24-h physical behaviors for better health. To promote health for all and reduce social gradient, we see a great need for empirically testing the ‘Sweet-Spot Hypothesis' with high-quality data and strong study design. We hope that the proposal of testing ‘Sweet-Spot Hypothesis' will encourage discussion, debates, and empirical research to expand our collective knowledge about the healthy ‘24-h physical behavior balance' for all.

